# A rat long‐lasting cystitis model induced by intravesical injection of hydrogen peroxide

**DOI:** 10.14814/phy2.13127

**Published:** 2017-02-27

**Authors:** Koji Dogishi, Ken Okamoto, Tsuyoshi Majima, Shizuka Konishi‐Shiotsu, Takashi Homan, Mizuki Kodera, Shohei Oyama, Tatsuya Oyama, Hisashi Shirakawa, Naoki Yoshimura, Takayuki Nakagawa, Shuji Kaneko

**Affiliations:** ^1^ Department of Molecular PharmacologyGraduate School of Pharmaceutical SciencesKyoto UniversitySakyo‐ku, KyotoJapan; ^2^ Discovery Research LaboratoriesNippon Shinyaku Co., Ltd.Minami‐ku, KyotoJapan; ^3^ Department of UrologyUniversity of Pittsburgh School of MedicinePittsburghPennsylvania; ^4^ Department of Pharmacology and Chemical BiologyUniversity of Pittsburgh School of MedicinePittsburghPennsylvania; ^5^ Department of Clinical Pharmacology and TherapeuticsKyoto University HospitalSakyo‐ku, KyotoJapan

**Keywords:** Chronic cystitis, hydrogen peroxide, hypersensitivity of bladder nerve, remodeling

## Abstract

Novel longer lasting inflammatory bladder animal models are needed to better understand the pathophysiology of chronic cystitis. We previously developed a relatively long‐lasting mouse cystitis model by intravesical injection of hydrogen peroxide (H_2_O_2_). To further evaluate its pathophysiology, in this study, we established and analyzed a rat cystitis model. Under anesthesia, 1.5% H_2_O_2_ solution was introduced transurethrally into the bladder of female rats, and kept for 30 min. The H_2_O_2_ injection significantly increased the number of micturition events up to day 14 and decreased urine volume per micturition, with the smallest volumes on day 3, compared with the vehicle‐treated group. Cystometric analysis on day 7 revealed that intercontraction intervals were significantly shortened without affecting the baseline, threshold, or maximum pressures. Intravesical resiniferatoxin‐evoked nociceptive behaviors, such as freezing, were significantly enhanced on days 7 and 14. Furthermore, histopathology revealed hemorrhage, edema, infiltration of neutrophils into the lamina propria, and urothelial denudation in the early phase (day 1). These damages were gradually repaired, while hyperplasia of the urothelium, vascularization, increases in fibroblast counts, and infiltration of mast cells and eosinophils were observed through the later phase (days 7 and 14). These results suggest that intravesical H_2_O_2_ injection induces relatively long‐lasting cystitis with enhanced bladder activity and pain sensation in rats. This approach thus provides a novel rat long‐lasting cystitis model that allows us to analyze detailed symptoms and pathophysiology of H_2_O_2_‐induced cystitis model than the mouse model and may be used to investigate the pathophysiology and treatment of chronic bladder hypersensitive disorders, such as bladder pain syndrome/interstitial cystitis.

## Introduction

Bladder pain syndrome/interstitial cystitis (BPS/IC) is a chronic inflammatory bladder disease defined by chronic pelvic pain that lasts for more than 6 months and pressure/discomfort accompanied by at least one other urinary symptom such as persistent urgency or urinary frequency (van de Merwe et al. 2008). Although epidemiological and experimental evidence suggests that BPS/IC may be caused by toxic urinary agents, inflammation, infiltration of mast cells, an autoimmune response, urothelial dysfunction, or neurogenic causes (Hanno et al. 2011; Davis et al. 2014). However, its detailed pathogenesis remains unclear, and there are few effective therapies or drugs for BPS/IC, which decreases the quality of life of patients.

To better understand the pathophysiology of BPS/IC, chronic cystitis animal models are needed. However, many experimental cystitis rodent models, induced by a single intraperitoneal or intravesical injection of ifosfamide, acrolein, mustard oil, or lipopolysaccharide/protamine sulfate, exhibit inflammatory responses and urinary symptoms for a relatively short period (several days) (Stein et al. 1996; Ribeiro et al. 2002; Batista et al. 2007). One popular model, the cyclophosphamide‐induced cystitis rodent model, is induced with a single intraperitoneal injection of cyclophosphamide, which induces acute hemorrhagic cystitis accompanied by acute inflammation, bladder overactivity, and pain‐related behaviors (Cox 1979; Olivar and Laird 1999). Repeated administration of low‐dose cyclophosphamide induces “chronic” cystitis, but this model is more appropriately considered to be a “repetitive acute” cystitis model (Boudes et al. 2011).

We previously established a long‐lasting cystitis model with an intravesical injection of hydrogen peroxide (H_2_O_2_) into female mice (Homan et al. 2013; Dogishi et al. 2015). A single intravesical injection of H_2_O_2_ induced relatively long‐lasting bladder inflammation and hyperactivity (>7 days post injection). The initial, severe urothelial damage and hyperpermeability caused by the H_2_O_2_ resolved within several days, while they could trigger long‐lasting bladder inflammation and hyperactivity, accompanied by vascularization in the submucosa and hyperplasia of the urothelium (Homan et al. 2013). This mouse model showed delayed and long‐lasting pain behavior, such as bladder distension‐evoked licking of the lower abdomen (Dogishi et al. 2015). These findings suggested that the H_2_O_2_‐induced mouse cystitis model would be a useful long‐lasting cystitis model. Experimental mouse disease models hold many advantages, including the potential to investigate molecular processes using genetically modified mice. Nevertheless, to study urodynamics and to conduct detailed behavioral and histological analyses, rat cystitis models are more appropriate. In this study, we developed a rat cystitis model by administering a single intravesical injection of H_2_O_2_ and performed behavioral analyses of micturition and nociceptive behaviors, cystometry, and histopathological examination of the bladder.

## Materials and Methods

### Animals

Female Sprague–Dawley rats aged 8–9 weeks were purchased from Japan SLC (Hamamatsu, Japan). They were housed and bred in groups of two per cage in a room maintained at 24 ± 1°C and 35–75% relative humidity with an alternating 12 h light/dark cycle (the lights came on automatically at 8:00 a.m.). Food and water were freely given. All animal care and experimental procedures were in accordance with the ethical guidelines of the Kyoto University Animal Research Committee, the University of Pittsburgh institutional animal care and use committee, the Internal Regulations on Animal Experiments at Nippon Shinyaku Co., Ltd., which are based on the Law for the Humane Treatment and Management of Animals (Law no. 105, 1 October 1973, as amended on 1 June 2006), the National Institutes of Health guidelines, and the Guiding Principles for the Care and Use of Laboratory Animals, approved by The Japanese Pharmacological Society.

### H_2_O_2_‐induced rat cystitis model

An H_2_O_2_‐induced rat cystitis model was created as previously reported for mice (12), with minor modifications. Under 2–3% isoflurane (Pfizer, NY) anesthesia, a polyethylene tube (PE‐50; Clay‐Adams, Parsippany, NJ) was introduced into the bladder transurethrally and then the lower abdomen was pressed gently to withdraw urine. Next, 300 *μ*L of 1.5% H_2_O_2_ solution (Wako Pure Chemical Industries, Osaka, Japan) suspended in sterile saline was introduced into the bladder through the catheter. The H_2_O_2_ solution was drained from the bladder after 30 min by pressing the lower abdomen.

### Micturition behavior

The micturition behavior of rats was analyzed under conscious conditions (*i.e*., voluntary voiding behavior), as described previously (Ozawa et al. 1999). Briefly, rats were individually habituated to the experimental setup in a metabolic cage for 24 h. After that, urine output was monitored for 24 h using a PowerLab 16/30 model ML‐880 (AD Instruments, Milford, MA) data acquisition system connected to a GX‐200 electronic balance (Kensei Industry Co., Inc., Ibaraki, Japan) ad libitum. At 1, 3, 7, and 14 days post intravesical injection of saline or H_2_O_2_, conscious rats were placed in the metabolic cages again, and their urine output was monitored for 24 h. The results were analyzed with the LabChart8 software (AD Instruments). Urine weight was converted to volume on the assumption that 1 g was equivalent to 1 mL. The parameters evaluated included the number of micturition events and the average urine volume per micturition (mL) for 24 h, during the light phase (8:00–20:00), and during the dark phase (20:00–8:00).

### Cystometry

Conscious cystometry was performed as described previously (Matsumoto et al. 2010). Under 2–3% isoflurane anesthesia, a PE‐50 catheter was inserted into the bladder through the dome, and implanted 7 days after intravesical injection of H_2_O_2_. The catheter was connected to a pressure transducer with a PowerLab 16/30 ML‐880 and an infusion pump through a three‐way stopcock. Saline was continuously infused into the bladder at a rate of 0.04 mL/min. After the rats were placed in a Bollman‐type restraining device (KN‐326; Natsume Seisakusho, Tokyo) and acclimated for 3 h, cystometrograms were recorded continuously for 30 min. Intercontraction intervals (ICI), baseline pressure (BP), threshold pressure (TP), and maximum pressure (MP) were analyzed with LabChart8.

### Resiniferatoxin (RTX)‐evoked nociceptive behaviors

The measurement of resiniferatoxin (RTX)‐evoked nociceptive behaviors was conducted as previously reported (Saitoh et al. 2008). Briefly, rats were acclimated in a plastic cage (Nalgene, Rochester, NY) for 2 h. Under 2–3% isoflurane anesthesia, a PE‐50 catheter was inserted into the bladder through the urethra, and the lower abdomen was compressed gently until the urine was withdrawn. The rat was then placed in a Bollman‐type restraining device. After the rat woke up, 300 *μ*L of RTX (3 *μ*mol/L), a transient receptor potential vanilloid 1 (TRPV1) agonist, or vehicle (10% ethanol, 10% Tween 80, and 80% sterile saline) was instilled into the bladder via the catheter and retained for 1 min. The transurethral catheter was then removed, and the rat was placed back into the plastic cage. Two behaviors, licking (licking to the lower abdomen) and freezing (motionless and face toward the lower abdomen), were scored every 1 min for 15 min in 5 sec intervals. When licking or freezing was observed during a 5 sec interval, it was scored as 1 point. The points were summed for the 15 min observation period to assign a total behavior score.

### Histological examination

Rats were anesthetized with sodium pentobarbital (64.8 mg/kg; Kyoritsu Seiyaku Co., Tokyo). Bladders were removed, fixed in 10% neutral buffered formalin, and embedded in paraffin. The paraffin‐embedded tissues were cut into 4 *μ*m sections and then stained with hematoxylin and eosin (HE) per standard procedures. Histopathological examination was performed with a light microscope (BX‐53F; OLYMPUS, Tokyo). Infiltrated cell types, such as mast cells, neutrophils, eosinophils and lymphocytes, were identified by their distinct morphology based on HE staining by a trained observer.

### Statistical analysis

All data are presented as the mean ± S.E.M. The statistical significance was assessed using a Student's t‐test, a two‐way analysis of variance (ANOVA), or a two‐way ANOVA for repeated measures, followed by Bonferroni post hoc test. The results were analyzed with the Prism 6 software (GraphPad, San Diego, CA).

## Results

### Micturition parameters in H_2_O_2_‐injected rats

**Figure 1:**
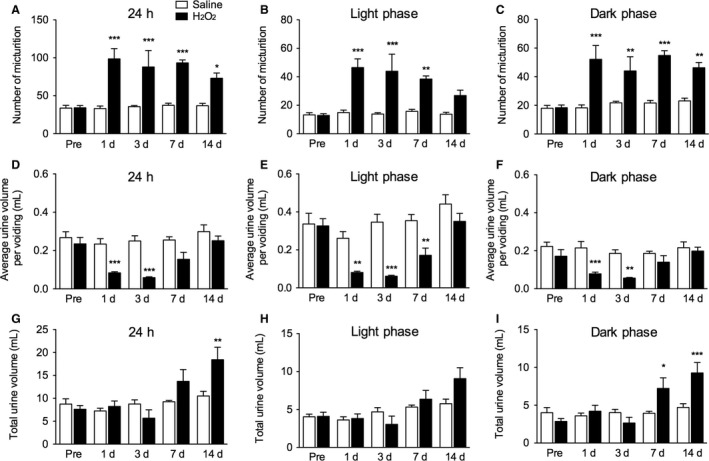
Micturition behavior in H_2_O_2_‐injected rats. Rats were injected intravesically with saline or 1.5% H_2_O_2_. At 1, 3, 7, and 14 days after the injection, the number of micturition events (*A–C*), the average urine volume per micturition (*D–F*) and the total micturition volume (*G–I*) were measured for 24 h (*A*,* D, G*), 12 h light phase (*B*,* E, H*), and 12 h dark phase (*C*,* F, I*). Values represent means ± S.E.M. for groups of seven rats. *P <* *0.05, **P <* *0.01, ***P <* *0.001, compared with the saline‐injected group.

The effects of an intravesical injection of H_2_O_2_ on the frequency of micturition and urine volume per micturition were examined 1, 3, 7, and 14 days after injection. The H_2_O_2_‐injected group exhibited significantly more micturition events over 24 h than the saline control group (*F*_1,68_ = 55.1, *P *<* *0.001). The difference began 1 day after injection and lasted for 14 days. Micturition was more frequent during both light and dark phases (*F*_1,68_ = 42.9, *P *<* *0.001 and *F*_1,68_ = 52.2, *P *<* *0.001, respectively). However, the difference in the light phase disappeared at 14 days, while more frequent micturition in the dark phase persisted for 14 days after the injection (Fig. 1A–C).

The urine volume per micturition over 24 h was significantly lower in the H_2_O_2_‐injected group than the saline control group (*F*_1,68_ = 39.2, *P *<* *0.001). The difference was observed 1 and 3 days after the injection. Although urine volume per micturition in the light phase was greater than during the dark phase, it was significantly decreased in both phases in the H_2_O_2_‐injected group (*F*_1,68_ = 39.1, *P *<* *0.001 and *F*_1, 68_ = 24.7, *P *<* *0.001, respectively). The significant decreases in the dark phase were observed at 1 and 3 days, while those in the light phase were seen at 1, 3, and 7 days after the injection (Fig. 1D–F).

However, the total micturition volume over 24 h was significantly increased in the H_2_O_2_‐injected group than the saline control group (*F*_1,68_ = 4.05, *P *<* *0.05), although the difference was observed only 14 days after the injection. The total micturition volumes in the light phase was not changed (*F*_1,68_ = 1.51, *P *=* *0.2234), while that in the dark phase was significantly increased in the H_2_O_2_‐injected group than the saline control group (*F*_1,68_ = 6.30, *P *<* *0.05). The significant increases in the dark phase were observed at 7 and 14 days after the injection (Fig. 1G–I).

### Cystometry in H_2_O_2_‐injected rats

**Figure 2:**
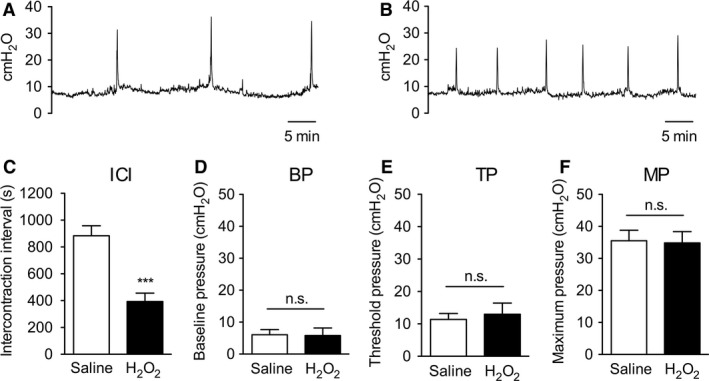
Cystometric parameters in H_2_O_2_‐injected rats. Rats were injected intravesically with saline or 1.5% H_2_O_2_. Cystometry was performed 7 days after the injection. Representative cystometrograms of saline‐ (*A*) or H_2_O_2_‐injected rats (*B*). Cystometric parameters included intercontraction intervals (*C*; ICI), baseline pressure (*D*; BP), threshold pressure (*E*; TP), and maximum pressure (*F*; MP). Values represent means ± S.E.M. for groups of 5–6 rats. ***P <* *0.001, compared with the saline‐injected group.

The effects of an intravesical injection on cystometric parameters were examined 7 days after the injection (Fig. 2). ICI was significantly lower in the H_2_O_2_‐injected group than the saline‐injected group. Other cystometric parameters, such as BP, TP, and MP, did not exhibit significant differences.

### RTX‐evoked nociceptive behaviors in H_2_O_2_‐injected rats

**Figure 3:**
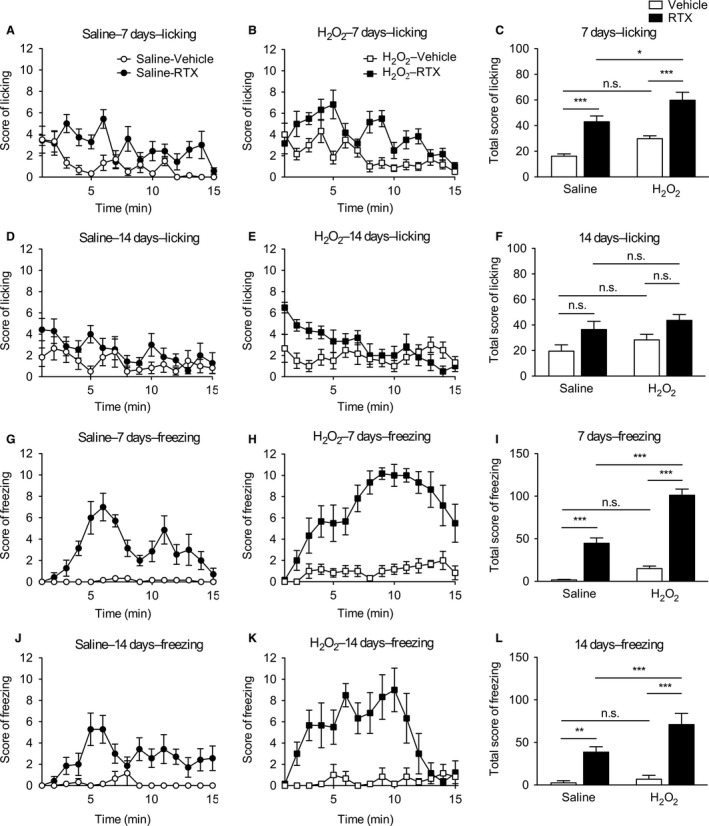
RTX‐evoked nociceptive behaviors in H_2_O_2_‐injected rats. Rats were injected intravesically with saline or 1.5% H_2_O_2_. RTX (3 *μ*mol/L) or vehicle was instilled intravesically 7 and 14 days after the injection, and then licking and freezing behaviors were scored every 1 min for 15 min (left and middle panels). Licking and freezing scores were summed over the full 15 min (right panels). (*A–F*) Licking score following vehicle or RTX instillation at 7 (*A–C*) and 14 days (*D–F*) post injection of saline (*A*,* D*) or H_2_O_2_ (*B*,* E*). (*G–L*) Freezing score following vehicle or RTX instillation at 7 (G–I) and 14 days (*J–L*) post injection of saline (*G*,* J*) or H_2_O_2_ (*H*,* K*). Values represent means ± S.E.M. for groups of 6–7 rats. *P <* *0.05, **P <* *0.05, ***P <* *0.001. n.s. = not significant.

The effects of an intravesical injection of H_2_O_2_ on RTX‐evoked nociceptive behaviors were examined 7 and 14 days after the injection (Fig. 3). The intravesical instillation of RTX increased the frequency of nociceptive behaviors such as licking and freezing. The RTX‐induced licking score was significantly higher 7 days after the injection (*F*_1,21_ = 47.1, *P *<* *0.001) in both saline‐ and H_2_O_2_‐injected groups as compared with that in the vehicle‐instilled group. The licking score was significantly larger 7 days after the H_2_O_2_ injection (*F*_1,21_ = 13.6, *P *<* *0.01). RTX‐evoked licking was significantly more frequent in the H_2_O_2_‐injected group than the saline‐injected group, although the licking score following vehicle instillation was slightly, but not significantly, increased (Fig. 3A–C). Similarly, a significant increase in RTX‐induced licking was observed at 14 days (*F*_1,21_ = 9.32, *P *<* *0.01), although a post hoc test revealed no significant difference between vehicle‐ and RTX‐instilled groups. H_2_O_2_ injection had no effect on licking behavior following vehicle or RTX instillation 14 days after the injection (*F*_1,21_ = 2.31, *P *=* *0.143; Fig. 3D–F).

The RTX‐induced freezing score was significantly higher 7 and 14 days after the injection (*F*_1,21_ = 158.8, *P *<* *0.001 and *F*_1,21_ = 42.7, *P *<* *0.001, respectively) in both saline‐ and H_2_O_2_‐injected groups as compared with that in the vehicle‐instilled group. The freezing score was significantly larger 7 and 14 days after the H_2_O_2_ injection (*F*_1,21_ = 46.1, *P *<* *0.01 and *F*_1,21_ = 5.75, *P *<* *0.05, respectively). Although the freezing score following vehicle instillation was slightly, but not significantly, increased, RTX‐evoked freezing was significantly more frequent in the H_2_O_2_‐injected group 7 and 14 days after the injection as compared with the saline‐injected group (Fig. 3G–L).

### Histopathological analysis of the bladders of H_2_O_2_‐injected rats

**Figure 4:**
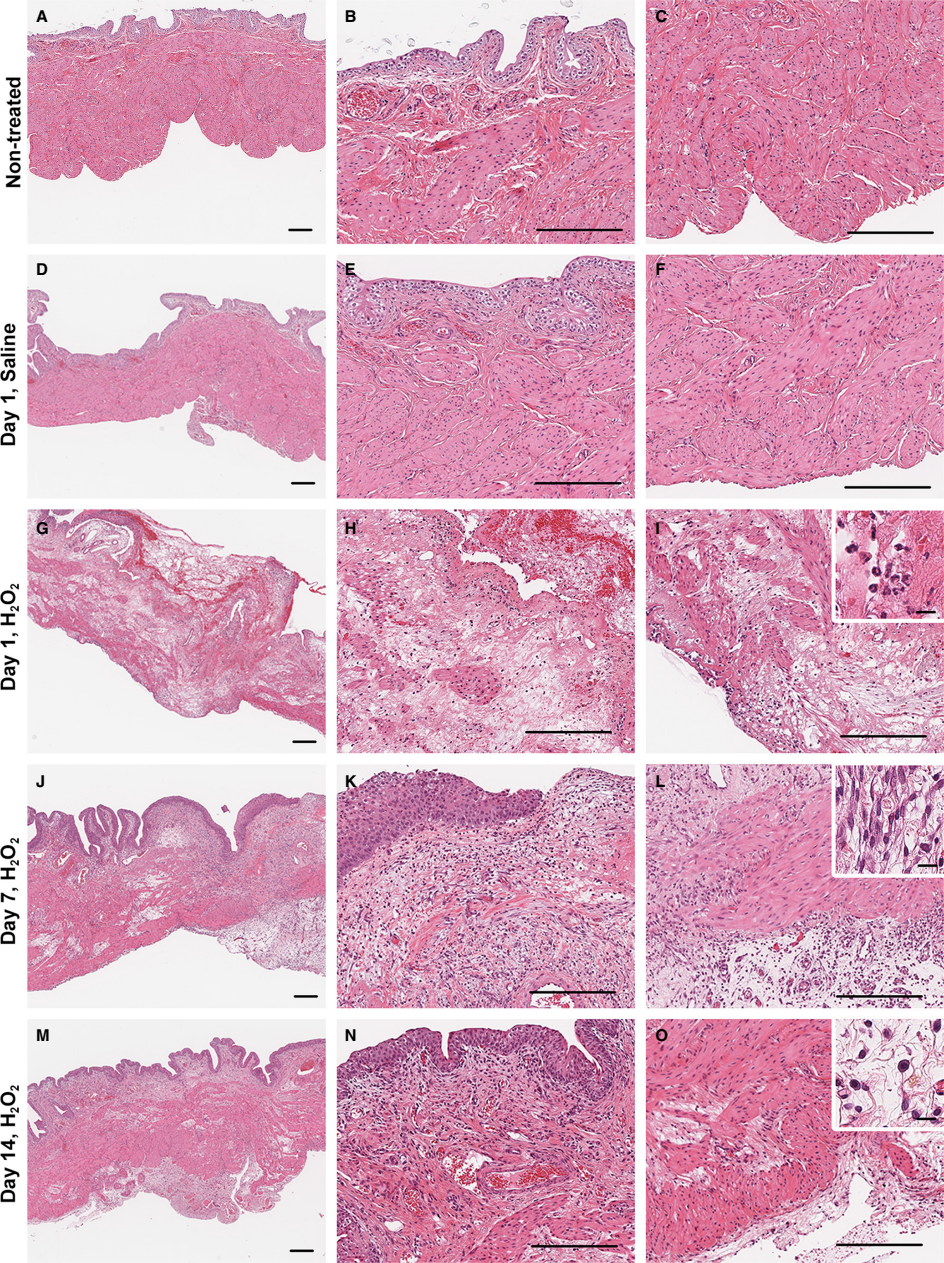
Histopathological changes of bladders in H_2_O_2_‐injected rats. Bladder sections were taken from untreated rats (*A–C*), or rats injected with saline 1 day after injection (*D–F*), or 1.5% H_2_O_2_ at 1 (*G–I*), 7 (*J–L*), or 14 days (*M–O*) after the injection, and examined by HE staining. Left panels show the low‐power field images of the bladder (*A*,* D*,* G*,* J*, and *M*). Central panels show the high‐power field images from the urothelium to the lamina propria (*B*,* E*,* H*,* K*, and *N*) corresponding to each left image. Right panels show the high‐power field images from the lamina propria to the smooth muscle layer (*C*,* F*,* I*,* L*, and *O*) corresponding to each left image. Bars = 200 *μ*m. Each inset shows enlarged images (*I*; neutrophils, *L*; fibroblasts, *O*; mast cells). Bars in enlarged images = 10 *μ*m.

Cystitis induced by the intravesical injection of H_2_O_2_ was histopathologically examined by HE staining (Fig. 4). Compared with non‐treated rats (Fig. 4A–C), no change was observed in the bladder tissue of saline‐injected rats (Fig. 4D–F). In H_2_O_2_‐injected rats, pronounced temporal changes in the bladder tissues were observed. On day 1, hemorrhage and edema were observed (Fig. 4G–I), and a large number of neutrophils infiltrated into the area from the lamina propria to the adventitia (Fig. 4I, inset). Furthermore, the smooth muscle layer was intermittently ruptured and denudation of the urothelium was observed on day 1. These changes, albeit weak, were observed even on days 7 and 14 (Fig. 4J–O). Mast cells were infiltrated into the lamina propria and smooth muscle layer on day 1, and this effect lasted until day 14 (Fig. 4O, inset). In addition, vascularization in the lamina propria and adventitia was observed on days 7 and 14 (Fig. 4J–L). A large number of fibroblasts were observed in the lamina propria (Fig. 4L, inset). Furthermore, a small number of infiltrated eosinophils and lymphocytes were observed from the lamina propria to the adventitia on days 7 and 14. By contrast, the denuded urothelium was repaired and then thickened, leading to hyperplasia on days 7 and 14 (Fig. 4K and N, respectively).

## Discussion

In this study, we developed a H_2_O_2_‐induced rat cystitis model, which shows relatively long‐lasting bladder inflammation, overactivity, and pain. This rat model has some advantages over the mouse model, allowing for more detailed study of the symptoms and pathophysiology of chronic cystitis.

To assess the micturition behavior of mice, we quantified the number of voids by counting urine spots on a filter paper after 15 min (Homan et al. 2013). However, the detailed analysis of micturition behaviors over a 24‐h period separately divided into light and dark phases is limited in the mouse model. In the current study of rats, we were able to analyze voluntary micturition behavior over 24 h using metabolic cages. Consistent with the H_2_O_2_‐induced mouse cystitis model (Homan et al. 2013), an intravesical H_2_O_2_‐injection increased the frequency of micturition in rats. This increased micturition frequency lasted up to 14 days in the rats, while it had disappeared within 14 days in mice. This suggests that cystitis can persist in rats for a longer period than in mice. Using metabolic cages also provided data on urine volume per micturition event, which dropped to its lowest level at 3 days and gradually recovered. Thus, the H_2_O_2_‐induced rat cystitis model shows increased frequency of micturition with smaller urine volumes in the early phase, while in the late phase (7–14 days) urine volumes recover even as micturition frequency remains high. These behavioral findings are further supported by the cystometric evaluation, which showed a reduction in ICI on day 7. Thus, the H_2_O_2_‐induced rat cystitis model exhibits persistent bladder overactivity because of hypersensitivity of the bladder sensory pathways, rather than the alteration of bladder distensibility or contractility.

Micturition behaviors are generally more frequent in the dark (wake) phase than in the light (sleep) phase in rats, because they are nocturnal animals. When the micturition behaviors were analyzed by phase, higher micturition frequency and smaller urine volume were observed in both light and dark phases, consistent with the clinical finding that BPS/IC and overactive bladder patients often experience increased day and night‐time urinary frequency (Kim et al. 2014). However, the increased number of micturition, at least, in 7 and 14 days dark phase may be due to the increase in the urine product, because total micturition volume at that time was increased.

Intravesical instillation of RTX evokes two nociceptive behaviors in rats, licking and freezing (Lecci et al. 1994). TRPV1 agonist‐evoked licking behavior is mainly mediated through the activation of urethral afferents in the pudendal nerve, rather than by the activation of bladder afferents in the pelvic nerve (Lecci et al. 1994; Saitoh et al. 2008). By contrast, RTX‐evoked freezing behavior in rats is characterized as a typical nociceptive response to activation of pelvic nerve afferents innervating the bladder (Saitoh et al. 2008). Gene transfer of anti‐inflammatory cytokines to the bladder and bladder afferent pathways reduces RTX‐evoked freezing without affecting licking in rats (Funahashi et al. 2013; Oguchi et al. 2013). Thus, RTX‐induced freezing is thought to reflect a pain response derived from nociceptive stimulation of the bladder. However, our preliminary experiments showed that it is difficult to observe RTX‐evoked licking and freezing separately in the H_2_O_2_‐induced mouse cystitis model (Dogishi et al. 2015). In this study, the present rat model allowed us to analyze the two behaviors separately. RTX‐evoked licking behavior was enhanced on day 7 in the H_2_O_2_‐induced rat cystitis model, although it disappeared in the later phase (14 days). By contrast, RTX‐evoked freezing was markedly enhanced on both day 7 and 14. These findings suggest that both urethral and bladder pain mediated through hypersensitivity of afferent nerves carried through pudendal and pelvic nerves, respectively, are induced in the H_2_O_2_‐induced rat cystitis model, and that bladder pain behavior can persist longer than urethral pain behavior. Accumulating evidence suggest that TRPV1 plays an important role in bladder overactivity and hypersensitivity in cystitis (Andersson et al. 2010). TRPV1 deficiency or selective antagonists suppress bladder overactivity and noxious behaviors in cystitis models (Charrua et al. 2007, 2009; Wang et al. 2008; Dornelles et al. 2014). The function of TRPV1 in bladder sensory neurons is enhanced in rat cyclophosphamide‐induced cystitis model (Dang et al. 2013). Consistently, increased severity of inflammation correlates with upregulation of TRPV1 in bladder nerve fibers of BPS/IC patients (Liu et al. 2014). These findings suggest that enhanced function or upregulation of TRPV1 in bladder sensory neurons may be responsible for the increased RTX‐evoked nociceptive behaviors in the H_2_O_2_‐induced rat cystitis model.

The histological analyses for the bladder of the H_2_O_2_‐induced rat cystitis model showed a variety of temporal changes in the bladder, consistent with the mouse cystitis model (Homan et al. 2013). In the early phase (day 1), severe histological damages, such as hemorrhage, edema, urothelial denudation and intermittent rupture of smooth muscle layer, and early infiltration of inflammatory cells, such as neutrophils and mast cells, were observed. We previously reported that intravesical injection of H_2_O_2_ causes rapid and short‐term hyperpermeability of the urothelial barrier in the mouse model (Homan et al. 2013). The acute damages to the urothelium can expose the lamina propria to irritants in urine, which may further exacerbate acute inflammation. Consistently, numerous neutrophils are observed in the urothelial blood vessels and the submucosal connective tissue in biopsies from BPS/IC patients (Saini et al. 2008). The initial damages were gradually repaired, but they persisted for a long time. In particular, infiltration of mast cells increased until the late phase (day 14) in the rat cystitis model, consistent with clinical observations indicating that the number of activated mast cells is increased throughout the bladder in BPS/IC patients (Sant and Theoharides 1994; Saini et al. 2008). Activated eosinophils are also reported in the urine of BPS/IC patients (Bouchelouche et al. 2001). We previously reported that inflammatory cytokines, such as tumor necrosis factor‐*α* and interleukin‐1*β*, are continuously upregulated in the bladder of the H_2_O_2_‐induced mouse cystitis model (Homan et al. 2013). This suggests that prolonged inflammatory responses triggered by H_2_O_2_‐mediated acute injury to the bladder are responsible for the hypersensitivity of the bladder and urethral sensory nerves, which leads to long‐lasting bladder overactivity and pain (Ghoniem et al. 2011; Funahashi et al. 2013; Oguchi et al. 2013).

Hyperplasia of the urothelium, vascularization, and an increase in the number of fibroblasts, *i.e*., fibrosis, were induced in the late phase (day 7–14), indicative of bladder remodeling that is consistent with observations from the mouse cystitis model (Homan et al. 2013). Bladder remodeling, including hypertrophy and fibrosis, is well documented in the bladder outlet obstruction model (Duan et al. 2015). Several lines of evidence suggest that obstruction‐associated decreases in blood flow and hypoxia in the bladder wall are associated with remodeling, which alters the bladder's contractility (Greenland et al. 2000). Although it has not been determined whether decreased blood flow and hypoxia are involved in the bladder remodeling of H_2_O_2_‐induced cystitis rodent models, the present findings suggest that chronic inflammation can induce bladder remodeling, which may contribute to long‐lasting bladder overactivity and pain. The bladder remodeling is not observed in other short‐lasting rodent cystitis models. Therefore, H_2_O_2_‐induced cystitis rodent models may provide novel evidence for bladder remodeling observed in BPS/IC patients (Richter et al. 2010).

In conclusion, the present study revealed that a single intravesical injection of H_2_O_2_ in rats induces relatively long‐lasting bladder inflammation and subsequent remodeling, which may contribute to elevated urination frequency and hypersensitivity of the bladder nerve. Thus, the H_2_O_2_‐induced rat cystitis model allows us to analyze more detailed symptoms and pathophysiology of the H_2_O_2_‐induced cystitis model, and presents symptoms and pathophysiology that are, at least in part, consistent with the characteristics of chronic cystitis observed in patients with BPS/IC. It is clearly necessary to establish novel chronic animal models showing longer lasting cystitis for a time frame of months. Nevertheless, this rat model, in combination with our mouse model, may be utilized to further investigate the pathophysiology and treatment of chronic bladder hypersensitive disorders such as BPS/IC.

## Conflict of Interest

The authors declare no competing financial interests.
